# Deaths in Buffalo in May, 1867

**Published:** 1867-06

**Authors:** Sandford Eastman

**Affiliations:** Health Physician


### Deaths in Buffalo in May, 1867.

Sex—Males, 57; Females, 52. Causes of death—asthma, 2; bronchitis, 2; cancer of the womb, 8; cholera morbus, 2; cirrhosis of liver, 2; consumption, 36; convulsions, 14; croup, 2; diphtheria, 6; dropsy general, 2; puerperal fever, 4; typhoid fever, 14; typhus fever, 2; gangrene, 2; inflamation of liver, 4; inflamation of lungs, 8; inflamation of lungs and typhoid, 4; inflamation of lungs and pleura, 2; inflamation of peritonium, 2; inanition, 4; kidneys, Bright's disease, 4; meningitis cerebral spinalis, 12; marasmus, 2; scrofula, 2; stricture of urethra, 2; syphilis, 2; unknown, 6; whooping cough, 2. Whole number of deaths 110.

Sandford Eastman, Health Physician.

